# Translation of surface electromyography to clinical and motor rehabilitation applications: The need for new clinical figures

**DOI:** 10.1515/tnsci-2022-0279

**Published:** 2023-03-14

**Authors:** Roberto Merletti, Federico Temporiti, Roberto Gatti, Sanjeev Gupta, Giorgio Sandrini, Mariano Serrao

**Affiliations:** LISiN, Department of Electronics andTelecommunications, Politecnico di Torino, Torino, 10138, Italy; Physiotherapy Unit, Humanitas Clinical and Research Center - IRCCS, Rozzano, Milano, 20089, Italy; Department of Biomedical Sciences, Humanitas University, Pieve Emanuele, Milano, 20090, Italy; Faculty of Allied Health Sciences, Manav Rachna International Institute of Research and Studies, Faridabad, Haryana, 121004, India; Department of Brain and Behavior Sciences, University of Pavia, Pavia, 27100, Italy; Department of Medical and Surgical Sciences and Biotechnologies, Sapienza University of Rome, Latina, 04100, Italy

**Keywords:** surface EMG, education, dissemination, physiotherapy, medical technology

## Abstract

Advanced sensors/electrodes and signal processing techniques provide powerful tools to analyze surface electromyographic signals (sEMG) and their features, to decompose sEMG into the constituent motor unit action potential trains, and to identify synergies, neural muscle drive, and EEG–sEMG coherence. However, despite thousands of articles, dozens of textbooks, tutorials, consensus papers, and European and International efforts, the translation of this knowledge into clinical activities and assessment procedures has been very slow, likely because of lack of clinical studies and competent operators in the field. Understanding and using sEMG-based hardware and software tools requires a level of knowledge of signal processing and interpretation concepts that is multidisciplinary and is not provided by most academic curricula in physiotherapy, movement sciences, neurophysiology, rehabilitation, sport, and occupational medicine. The chasm existing between the available knowledge and its clinical applications in this field is discussed as well as the need for new clinical figures. The need for updating the training of physiotherapists, neurophysiology technicians, and clinical technologists is discussed as well as the required competences of trainers and trainees. Indications and examples are suggested and provide a basis for addressing the problem. Two teaching examples are provided in the Supplementary Material.

## 1 Introduction

### 1.1 Background and state of the art

The analysis of skeletal muscles through the sEMG is fundamental for understanding the organization and production of movement. Furthermore, it provides extensive information for medical doctors, physiotherapists, occupational therapists, and movement scientists for functional diagnosis, patient path management, evaluation of patient recovery and progress, and quantification of treatment/training effectiveness. Most clinical studies are limited to few subjects (usually <30) and look more like “proof of concept” studies. Larger clinical studies require funding and competent operators and both are lacking. This work addresses the need for training clinical operators in the use of sEMG as a measurement tool.

The milestone book by Basmajian and De Luca [1] provided the first truly multidisciplinary approach for teaching sEMG using basic signal processing concepts in addition to the anatomical and neurophysiological knowledge. The complexity of the sEMG signal, consequent to the large amount of information contained in it, motivated the work of many investigators aiming at the extraction of such information. On one hand, this effort generated many mathematical approaches (not all appropriate for the task or understandable by the users), as described by Hodges and Bui since 1996 [2] for the case of determining the onset of muscle contractions. On the other hand, this effort generated a large number of technical publications that created confusion among the potential users. Despite the efforts of De Luca, Kasman, Cram, and many others [1,3,4,5], a gap opened between technical knowledge and clinical applications by users lacking this knowledge. Extensive publication of mathematical and engineering content established perception of sEMG as a high-tech research instrumentation demanding advanced mathematical expertise. This placed sEMG at a disadvantageous position as there has been an inherent tendency, still persisting in clinician’s mindset, to avert mathematical analysis. The need for a knowledge translational effort became evident among the engineering community in the early 80s and is still felt in the clinical fraternity.

In 1999, the European Project “Surface Electromyography for Non Invasive Assessment of Muscles” (SENIAM) provided a set of Recommendations for sEMG. These were described in seven booklets plus a summary volume, a CD ROM, and a website [6] (www.seniam.org, now also available in www.robertomerletti.it/en/emg/material/seniam/). The project involved partners from nine EU countries and provided guidelines about electrodes and their placement, signal processing, modeling techniques, and data reporting. Despite their frequent citation in many papers, and their recent updates [7,8], these guidelines have neither been respected in many works nor taught in most academic courses in the fields of rehabilitation, movement sciences, ergonomics, and occupational medicine.

**Figure 1:**
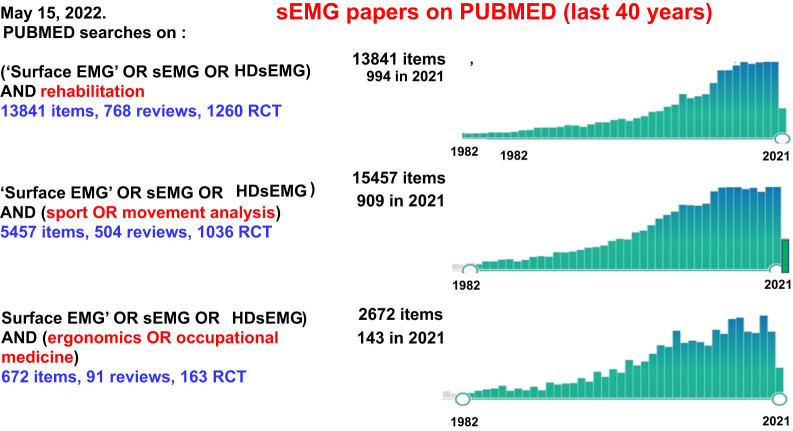
Results of three searches on PubMed (May 15, 2022) indicating the number of publications, reviews, and randomized control trials in the last 40 years, mentioning or using sEMG in the fields of rehabilitation, sport or movement sciences, ergonomics or occupational medicine.

In the last 20–30 years, the sEMG technology underwent huge advances resulting from the development of 2D electrode arrays, wireless signal transmission, and techniques for extracting anatomical and neurophysiological information. The continued developments in technology of sEMG were not complemented with proportionate penetration of sEMG in its targeted clinical uses. In two to three decades, potential applications of sEMG extended from the traditional physiotherapy field to neuroscience, obstetrics, ergonomics, and many other areas [9,10]. While the number of scientific publications increased (see Figure 1), dissemination, teaching and clinical use lagged very much behind. When considering such inertia in terms of dissemination, teaching and clinical use of sEMG, the poor spread of practice-based research approaches should be considered [11].

The International Society for Electromyography and Kinesiology (ISEK, www.isek.org) recently promoted two translational initiatives through its *Journal of Electromyography and Kinesiology* (*JEK*, https://isek.org/isek-journal/). The first is a series of tutorials aimed to readers with limited engineering background. Three of these tutorials have been published and a fourth is in preparation [7,8,12]. The second initiative is the “Consensus for Experimental Design in Electromyography” (CEDE project) providing a number of consensus papers on sEMG-relevant topics [13,14,15,16,17]. They provide excellent teaching material, prepared by experienced MDs, physiotherapists, movement scientists, and engineers. Another translational initiative was promoted by “Sensors” with the Special Issue on “Advances in Bipolar and Array-Based Surface EMG: Detection, Interpretation and Teaching” (https://www.mdpi.com/journal/sensors/special_issues/Teaching_sensors) and deals with teaching fundamental concepts of sEMG [18,19]. A translational effort was promoted by J. Pons and associates with the organization of the International Conference of Neuro-Rehabilitation (ICNR) and the Summer School on Neuro-Rehabilitation (SSNR) [20]. In addition, a set of 10 open access bilingual (English and Italian) teaching modules on sEMG (with no mathematics) is freely available from https://www.robertomerletti.it/en/emg/material/teaching/.

Despite these initiatives, many barriers remain as factors limiting the widespread clinical use of sEMG and the gap between technological possibilities and their clinical applications is widening. This situation has been recently addressed by the open book “Surface Electromyography: Barriers limiting widespread use of sEMG in clinical assessment and neurorehabilitation” containing 18 contributions on this issue and freely available from: https://www.frontiersin.org/research-topics/11157. This editorial initiative identified the barriers as mainly (1) cultural, (2) educational, (3) technical, and (4) administrative [10,21,22].

The efforts of ISEK, journals, and individual researchers in translational initiatives aimed at clinical operators and their teachers are remarkable, but results are still far from target, as reported by Braun et al. [23] in 2018. While general fundamental issues are discussed in refs [11,24] and barriers are addressed in ref. [22] (https://www.frontiersin.org/research-topics/11157), this work addresses dissemination/education problems and training of new clinical figures. Specifically:

1. Insufficient competence in effective dissemination and in the analysis of failures. This is discussed (for EU engineering projects but is applicable to the sEMG field as well) by R. Suurla in the book “Methods and tools for effective dissemination” [25].
2. Insufficient education and training provided by the academic courses despite the interest of the students [26].
3. Excessive focus on research publications (mostly in engineering), which often encounter difficulties in being transferred into clinical practice by clinicians [27,28,29,30].
4. Lack of multi-pronged dissemination by way of “training the trainers,” brainstorming with policy makers, involvement of leaders of rehabilitation teams, no accredited/certified courses with standardized content in the field.
5. No direct formal linkages between the engineering community and associations of rehabilitation professionals with targeted sEMG promotion.
6. Cultural and educational barriers. General diffidence toward innovation and electronic measurement instrumentation [23].
7. Lack of awareness of the scientific literature and limited relevance of it in the educational process, local situations. This is an important issue discussed in the work of A. Jette [27,28] who states “*Local worlds rule the clinical networks in which we live and practice and have substantial influence on the clinical behaviour of rehabilitation professionals. This creates a clash of cultures that delays the adoption of practice innovations.*”

These issues are addressed also in refs [29,30] and will be further discussed in the following sections.

### 1.2 Generalities on knowledge translation channels

The difficulties in disseminating sEMG techniques are strongly connected to the broader issues of translation of information and scientific evidence. This is a priority in US Public Health [31,32]. In the last few decades, communication technology, advertisement, and social media enabled faster awareness of innovations (not necessarily on the basis of their scientific merits). Jippes et al. [33] examined the effect that an intensive training had on dissemination and assessing clinical competencies among medical specialists [34]. They concluded that “*We found a strong effect for network tie strength and no effect for the Teach-the-Teacher training course on the dissemination of the new structured feedback technique. This paper shows the potential that social networks have for disseminating innovations in health service delivery and organization…… the question then arises as to how effective training and education actually are in distributing and transferring novel ideas, new health concepts, and technologies.*” Although initiatives such as the *Teach-the-Teacher* training can improve didactic abilities and skills [35], this by itself is apparently not enough to adopt innovations.

The following pros and cons are well codified: cost and the perceived monetary advantages, time spent in learning, effectiveness, simplicity (or how easy is to learn and use the innovation), compatibility (or how well the innovation fits with established procedures), observability (or the extent to which outcomes can be seen), trialability (or the extent to which the adoption decision is reversible or can be managed in stages), social pressure to do so, and, most important, the judgments of trusted expert and opinion leaders. “*When opinion leaders do not adopt an innovation, systems do not change. Diffusion is an atypical outcome, since the vast majority of innovations fail to diffuse. Unworthy innovations sometimes diffuse, and effective innovations are often stymied*” [28]. Very likely many of these problems would be removed if the innovations where taught in schools and made mandatory to practice by statutory policy [36].

Helgøy et al. [37] recently published a scoping review on physiotherapy education programmes and suggested four strategies to reduce the translational gap: (1) student engagement in research, (2) curriculum improvement regarding evidence-based practice (EBP), and (3) EBP teaching and (4) journal clubs. “EBP teaching” was most frequently identified as a barrier. This and the “journal club” imply the teacher’s ability to lead the discussion of EPB and of the literature. This is not easy in countries where there are no PhD degrees or academic professorial positions in physiotherapy.

### 1.3 Generalities on sEMG use for clinical purposes

Measurement is the process of finding the size of a physical quantity by attaching a value expressed as a number of units (e.g. blood pressure in mmHg, torque in Nm, sEMG in Volt, etc.). This process requires a measuring instrument used by a competent person. Other “quantities”, such as pain, feelings, or mental processes are evaluated by means of analogue scales based on subjective reporting, usually between 0 and 10 where 0 means absence of “something” and 10 means maximum possible value of the same thing. Scale evaluations suffer from subjectivity and limited repeatability. Measurements and scale evaluations are the foundation of EBP in rehabilitation and are used to quantify the result of some intervention or treatment (outcome measures). The ability to manage measurements and scale evaluations is fundamental for clinical operators. The theory of measurements and of measuring instruments is an important part of education in physics and engineering, unlike in rehabilitation medicine where scales are generally used [23,38]. Some quantities and their measurements depend on the quantity’s definition. For example, can the “neural drive” to a muscle be measured? Can the association between EEG and sEMG be measured? Can synergies, tremor, degree of denervation, or other “quantities” be measured? What are their units? When and how a measurement should be “normalized” with respect to a reference value and expressed as a percentage of this value?

In the last two decades, the possibility of decomposing the sEMG into the constituent trains of motor unit action potentials opened a new window on measurements of the CNS. It is now possible to measure the discharge rate (the “neural drive”) of single detected MU in pulses/s and the force (or torque) in N or Nm at which an MU is recruited or de-recruited. The “neural drive” to the muscle (the number of the detected motor neuron action potentials impinging on the muscle every second) is more correlated to “intentional muscle force” than the sEMG amplitude (RMS value) and is associated to tremor and to the EEG–EMG coherence [39,40,41]. The operator performing these measurements must understand these processes and be aware of the effect of approximations, noise, interferences, and confounding factors, such as crosstalk. Therefore, he/she must have received a specific technical training in the fields of electrophysiology and signal processing and be fully aware of, and able to apply, these concepts to clinical cases [42,43].

Surface EMG is instrumental in a variety of applications including muscle physio-pathology, disease prevention, planning interventions, monitoring, and quantifying changes due to interventions, monitoring workplace, and sport activities. It is not replacing needle EMG, which is a medical diagnostic practice, and has a number of well-known advantages and disadvantages with respect to the surface technique [44]. Too often the diagnostic and therapeutic effects are underlined rather than assessment applications.

Although the situation is rapidly changing, the use of sEMG for diagnosing disorders is, in general, limited, with few exceptions related to gait analysis and fatigue assessment [44]. This is a consequence of the relevance that some confounding factors have in determining sEMG amplitude (thickness and electrical properties of the subcutaneous tissue, location of the muscle innervation zone, muscle fiber pinnation angle, etc.) [45]. Because of these factors, normalization of sEMG with respect to the value corresponding to the maximal voluntary contraction (MVC) is required to allow comparisons of sEMG amplitudes (as percentages of the MVC values) between different muscles and subjects [14]. For example, the fact that the sEMG of muscle A shows a greater amplitude than muscle B does NOT necessarily imply that muscle B has a higher “drive” or produces a greater force than muscle B since A may be more superficial, or the electrodes may be in better position than in the case of B. This fact is the source of many incorrect conclusions and is now being overcome with the observation that muscle force is more closely related to the cumulative spike train (global discharge rate) of the active MUs rather than to sEMG amplitude [46,47]. Like needle EMG, sEMG is used preferably in isometric contractions which provide controlled “bench test” conditions, not common in daily life activities. While the study of dynamic contractions is quite possible with sEMG, special caution must be used to make sure that the observed changes are due to physiological factors and not to geometrical changes [45]. This situation is often leading to sEMG misinterpretation by health operators. Therefore, it is important to ensure that (1) the collected “raw” EMG signals are of sufficient quality prior to further analysis, (2) the analysis is performed correctly, and (3) the operator is aware of the possible sources of error, multiple interpretations, misjudgments and possibly wrong conclusions consequent to improper use of the technique.

In summary, like in most other medical assessments procedures, the use of sEMG requires training, competence, experience, and awareness of limitations [15,26,48].

Novel applications in the assessment of neuromuscular disorders are being developed (e.g. in the suppression of essential tremor [41] and in Charcot–Marie–Tooth disease [49] among many others). As stated by Shahrizaila [50], sEMG is an important disease biomarker and “*we are just scratching the surface.*” Nevertheless, potential users and teachers too often shy away from the quantitative assessment and from the evidence-based rehabilitation potential of sEMG. For this reason, despite the huge amount of literature, most of the potential provided by sEMG in monitoring neuromuscular disorders remains unapplied. Authoritative studies focused only on the diagnostic limitations of classical bipolar sEMG methods [44] disregarding how high-density sEMG (HDsEMG) technology is opening up specific applications, such as (1) the estimation of the neural drive to a muscle, (2) the assessment of the coherence between EEG and EMG signals, and (3) the observation of the synergies used in programming and executing movements. These and other issues cannot be investigated with other current methodologies and, therefore, comparisons with alternative methods, as suggested by Meekins et al. [44], are not possible. These technological advances provide a perspective on (1) how the CNS controls motor unit activities, and therefore the forces exerted by muscles and (2) the modularity of such control by combining “synergies” [51,52]. A translational effort is needed to promote applications of these procedures in the clinical world following the many “proof of concept” studies [53,54,43,49,55,56,57,58].

## 2 Effective dissemination by teaching and research

In 2019, the US National Institute for Health and Care Research published a guide about dissemination of research results (https://www.nihr.ac.uk/documents/how-to-disseminate-your-research/19951). They acknowledged that one should “*be aware of the relevant current cultural and political climate*” and that “*dissemination might be perceived differently by different groups.*” Different groups may have different priorities and corporative interests. Cultural climate is important for successful dissemination of innovation and is mostly created in schools.

The report “Methods and tools for effective dissemination. A guide to the dissemination of the results of International Educational Projects” [25] recommended to involve the future users since the beginning of any innovation project but also admitted that failures can result from:

1. The target partners do not have the expertise necessary for utilization of results;
2. Bureaucratic and administrative difficulties slowed down the dissemination process;
3. Lack of financing after the end of the project;
4. Weak recognition of the importance of dissemination;
5. Lack of time to learn and competitive commitments of the partners/users;
6. The product is not market oriented;
7. Political and economic situation in the country(s) where dissemination was planned;
8. Partners with insufficient reputation/capability in the educational field;
9. Cultural and ideological differences between the partners too large; and
10. Expectations and cultural background of the target partners are too different.

Dissemination of sEMG through publication of new findings in scientific journals does not usually work. As A. Jette indicated “*Publishing our work in journals is essential but publication of research is not, by itself, sufficient if our goal is to change clinical practice. People follow the lead of other people they know and trust when they decide whether to take up an innovation and change the way they practice!*” [28]. Tutorials and consensus papers published by qualified scientific journals have an impact lower than that of “influencers” on the social media.

### 2.1 Dissemination by teaching

The most important form of dissemination is teaching. The role and responsibilities of undergraduate education have been underlined by Snöljung et al. [30] who reported the need to teach the use of measuring instruments at the undergraduate level. In agreement with many other studies, the survey of Scurlock-Evans et al. [29] concluded that “*Practitioners reported difficulties in reading journal articles and rated literature and research as low priorities for implementing best clinical practices*” and that barriers to EBP implementation become apparent as “*…lack of time and skills, misperception of EBP and of what constitutes evidence*” suggesting that EBP practices should be learned in school and not during the professional activity when time to do so is no longer available.

Many countries do not offer a PhD and academic career in physiotherapy. This leads to a lack of professors in the field and the need to either recruit professors from related fields or among practitioners, with yearly contracts. Both often lack, for different reasons, the required competence and teaching skills in the field, in particular in interdisciplinary areas dealing with technology, instrumentation, and signal processing. Unquestionably, there is a need to teach the teachers what to teach and how to teach it, considering the audience background. Only few teaching hospitals address the problem [34]. According to the survey done by Manca et al. [26] (35 authors of at least two articles concerning sEMG applications in peer-reviewed journals), five years of clinical experience are adequate to teach sEMG. Not many teachers satisfy this requirement today.

### 2.2 Dissemination by research

The fast development of rehabilitation and sEMG technologies implies a strong link between research and education of the clinical operators in the field. This implies a critical understanding of biomechanics and electrophysiology and not just the “blind” application of the few commercially available equipment [59]. The recent review by Helgøy et al. [37] analyzes 27 studies on research-based teaching concluding that “…*ensuring students’ competence in research methods is necessary for students to be able to read and understand research articles which are important foundational skills in undergraduate research training. Journal clubs can be a foundation for student engagement with research literature*…” and “…*research-based education should be increased among both faculty members and students.*”

In conclusion, better dissemination (mostly through better training of teachers) would increase familiarity with the concepts of measurement and reduce “*…the lack of capacity to transform research results into practice and the difficulty in reading or even accessing journals*” [30].

## 3 Why and by whom is sEMG processing and understanding needed?

A question frequently asked by students and rehabilitation professionals is “What is sEMG useful for?” This question reveals the need for a translational effort and has a wide range of answers that imply knowledge of the information that can be extracted from sEMG. It brings up a long list of sub-questions some of which are: how important is to evaluate the pattern of muscle activation, fatigue, or compensatory strategies following a lesion? How important is to evaluate muscle timing during walking, the features of single motor units, or the connectivity between brain and muscle signals? Is the evidence provided by sEMG welcome or disagreeable in planning interventions or assessing results? The list is much longer and relevant; some examples are reported in the following subsections to sustain the need to adopt a practice-based research model as solution to increase the sEMG use.

### 3.1 Examples of sEMG application: sEMG for arthrogenic muscle inhibition detection and rehabilitative implications

#### 3.1.1 Example 1

Motor recovery is sometimes incomplete following orthopedic surgery, where impairments in neuromuscular control often persist over time despite the complete restoration of functional independence. These impairments include (1) decreased neuromuscular activity due to arthrogenic muscle inhibition, (2) improper muscular activation timing and (3) underuse of motor potential of impaired body structures in favor of adaptive strategies involving unaffected segments. In this scenario, clinical examination of patients’ motor behavior can certainly take advantage of sEMG to detect the aforementioned impairments and plan appropriate rehabilitative interventions [60].

Arthrogenic muscle inhibition causes inability to fully activate a muscle and represents a common phenomenon in knee degenerative conditions or surgery, where decrease in quadriceps torque and sEMG activity have been reported [61]. Rehabilitative exercises, such as maximal strength training, are usually adopted to enhance quadriceps neuromuscular activity. Monitoring this activity through sEMG becomes fundamental to select the best training modality [62]. For example, Ruspi et al. investigated the neuromuscular activity of quadriceps bellies during three maximal isometric tasks (knee flexion, hip flexion and hip flexion while performing a contralateral hip extension) performed in the same biomechanical condition in patients before and after knee arthroplasty compared to healthy age-matched subjects. Patients revealed higher vastus medialis activation during hip flexion than during knee extension after surgery, unlike healthy subjects who showed higher vastus medialis activation during knee extension. These findings demonstrated the irreplaceable role of sEMG and provided useful indications for planning effective training [63].

### 3.2 Surface EMG to assess improper muscle activation timing and rehabilitative implications

#### 3.2.1 Example 2

Altered muscular activation patterns often occur in cases of functional independence in patients with musculoskeletal conditions or after orthopedic surgery, and the detection of such impairments requires sEMG signal collection and analysis. For example, studies reported prolonged quadriceps activity during the stance phase of gait cycle, leading to increased quadriceps-hamstring coactivation in patients after knee surgery [64,65]. Furthermore, increased quadriceps-hamstring coactivity has been reported during sit-to-stand from a chair and during stair descending, as a result of muscle weakness and joint instability [66,67]. Similarly, Hodges and Richardson adopted sEMG to describe a transversus abdominis neuromuscular activation delay during upper limb movements in subjects with low back pain, identifying this phenomenon as a marker of inefficient muscular stabilization of the lumbar spine and leading clinicians to develop ad-hoc exercise programs aimed at improving spine functional stability [68].

### 3.3 Surface EMG for the detection of adaptive strategies and rehabilitative implications

#### 3.3.1 Example 3

Functional recovery can occur either through the restoration of motor abilities of affected body structures or by means of adaptive strategies aimed at compensating the impaired body function. Adaptation has been largely described in patients with CNS lesions, but it also occurs after impairments of peripheral body structures. Clinical examination alone may not distinguish between motor recovery of the affected structures and adoption of adaptive strategies involving unaffected structures, especially in patients with complete functional recovery. For example, Temporiti et al. described asymmetries in body weight distribution during quiet standing in patients before and after unilateral hip arthroplasty. These patients relied on the unaffected limb to stabilize the whole body and ensure postural stability [69]. Adaptive strategies in patients undergoing lower limb orthopedic surgery usually increase during demanding tasks such as sit-to-stand, where lower internal knee extension torque at the level of the affected limb results in lower sEMG of the quadriceps [69,70]. In this context, sEMG may be crucial for assessment purposes and for planning proper rehabilitative intervention. Furthermore, sEMG biofeedback may be used to inhibit the unaffected limb muscles and their overuse during functional tasks.

### 3.4 Who should carry out sEMG measurements in clinical practice?

Patients undergoing a motor rehabilitation program are required to perform tailored therapeutic exercises, which are defined after an accurate functional assessment. In this scenario, sEMG is essential to provide such assessment as well as to show the presence of arthrogenic muscle inhibition, improper muscle activation timing, or the use of adaptive strategies [60]. The detection of these conditions provides a valuable support in the selection of the most appropriate therapeutic exercise. Therefore, experts in the detection, processing, and interpretation of sEMG must have a role in rehabilitative practice. It is worth noting that many clinical questions requiring an sEMG analysis are specific for each patient and cannot be standardized.

In this scenario, the professional(s) dedicated to sEMG in clinical practice (1) must be aware of, and be familiar with, the most updated and appropriate literature in the field and (2) must have a strong interdisciplinary medical-technical knowledge. What kind of education should these professionals receive? In general, the complexity of sEMG data analysis does not allow for the inclusion of the required skills in a 3 year Bachelor program and requires a 4 year curriculum (see, for example, https://collegedunia.com/courses/physiotherapy/syllabus).

Therefore, ad-hoc academic educational training, delivered at post-Bachelor academic courses and targeted to professionals involved in the rehabilitation team, should provide this kind of specialistic and multidisciplinary education. Alternatively, multidisciplinary teams should be taken into considerations (Section 4.3). The issue of teaching basic concepts about sEMG starting at the BS level while providing additional information at the MS level is still somewhat controversial and is discussed in the following section.

## 4 Education and training of professional operators

The following subsections consider two established types and a third recently proposed type of clinical operators and outline different options concerning these figures and their training. Other figures have been proposed such as the movement scientists or the kinesiologist in the sport field.

The Bologna Process (1999) established the European Higher Education Area to improve student and staff mobility, to make higher education more inclusive, accessible, appealing, and competitive. All participating countries agreed to implement a three-step higher education system consisting of Bachelor (BS), Master (MS), and Doctoral (PhD) levels. Given this organization, sEMG could be taught at the BS, MS, and PhD levels, as well as at postgraduate certified courses. At the BS level, the technical general knowledge of sEMG should be provided at least in basic Neural Engineering and Neuromuscular Physiology courses. The sEMG may be taught more deeply at the MS level to neurophysiology technicians and physiotherapists. The PhD courses (when available) offer a wide range of research programs in neurosciences, biomedical signal processing, and biomechanics and provide a background for teaching. Furthermore, the fundamental concepts of sEMG should be taught during the medical specialization courses in Neurology, Rehabilitation, and Orthopedics. There are other non-university high education programs (e.g. in Germany). Some National/International Scientific Societies provide certificates as well as the possibility to develop professional figures or promote dissemination of knowledge.

### 4.1 Physiotherapists and occupational therapists

Despite the current situation [23], physiotherapists are at the pivot of creating basic knowledge about new technologies in clinical contexts. The role of the physiotherapist is vital in the assessment, monitoring, and rehabilitation of all major movement disorders. With the recent phenomenal increase in technical knowledge, the physiotherapist is potentially placing itself at the most efficient lever to deliver evidence-based therapies in a rehabilitation team. A clear majority of physicians and surgeons endorse the role of physiotherapist as vital in early and effective functional restoration of movement and activity after most of the medical and surgical procedures. There is acceptance of physiotherapist in society and patient community at large. Being recognized as a pain relieving and activity restoration specialist by non-drug methods, physiotherapists are welcome saviors for managing chronic ailments. Although, for decades, physiotherapists have extensively exploited electronic instrumentation and technology for therapy (Electrical Stimulation, LASER, ultrasonic, and diathermies), they have not been trained in the field of measurement systems. Methods such as chronaxie, rheobase, and strength–duration curves are age old in physiotherapy practice but recent electrophysiological and biomechanical techniques, such as sEMG and Inertial Measuring Units (IMU), have not yet been introduced in their educational curriculum. Once properly trained, physiotherapists could effectively contribute in mustering required clinical aspects of sEMG knowledge and disseminate the potential of sEMG-based assessment. The precise role of physiotherapists in this regard can be categorized in the following strata:

1. Infrastructure requirement and curriculum (policy making): sEMG instrumentation should be included in physiotherapy labs (as done in India and many other countries), with standardized teaching in a 3 year BS curriculum. A dedicated subject in undergraduate education and in postgraduate programs is desirable, with proportionate weightage in examination and evaluation. This is a practical approach to avoid the inertia to adopt new technologies that is observed in experienced physiotherapists at later times.
2. Clinical practice: there should be a policy on “Standards of Procedures” to guide and enhance sEMG reporting during clinical assessment and monitoring of neuromuscular disorders. Physiotherapists should contribute to these standards.
3. Research thrust: sEMG research in MS and PhD programs (where available) may be promoted by offering grants, seed money, access to literature, fellowships, and idea incubation centers.
4. Faculty development programs should grant credits to attend sEMG-related workshops, conferences on evidence-based practices, etc. to disseminate sEMG techniques. Career enhancements should provide physiotherapists with promotional avenues in reward of exemplary practice, case studies, innovations, peer-reviewed publications, etc.

In summary, the current academic curriculum in physiotherapy is not suitable to produce a clinical figure with proper competence in sEMG and other assessment techniques. Its updating is long due and proper pressure and educational initiatives promoted by the professional societies are necessary. The recent initiatives of the ISEK are a good step in this direction (https://isek.org/isek-jek-tutorials/). Other specific “URLs” dealing with academic curricula are:

https://collegedunia.com/courses/physiotherapy/syllabus

https://physio.sgtuniversity.ac.in/syllabus-first-year-bachelor-of-physiotherapy-bpt/

https://www.getmyuni.com/bpt-syllabus-subjects

https://www.amsterdamuas.com/programmes/european-school-of-physiotherapy/programme-structure.

### 4.2 Neurophysiology technicians

One of the professional figures that would best suit the role of sEMG management is the “neurophysiology technician” (in India and other countries called “EMG Technician”). Professionals in the field of neurophysiology perform tests that assist physicians and surgeons in the diagnosis and evaluation of diseases of the brain, peripheral nervous system disorders, and sleep using sophisticated electronic testing equipment. According to the report of the European Commission (https://ec.europa.eu/growth/tools-databases/regprof/professions), this profession is well regulated in three countries across the European Union, notably in Hungary (Klinikai neurofiziológiai szakasszisztens), Italy (Tecnico di neurofisiopatologia), and Portugal (Técnico de neurofisiologia). In other countries, sEMG and EEG technicians are professional figures specifically trained within certificated and specific courses (US and UK). In Italy, the Law Decrees of January 26, 1988 and March 15, 1995 provided a clear legal identification of the neurophysiology technician professional profile within the National Health Service. Accordingly, the neurophysiology technician is a health professional figure who works in the field of neurology and neurosurgery, uses the necessary instruments (electroencephalography, electroneuromyography, evoked potentials, sleep polygraphy, and ultrasound), and is responsible for their operation and for proper bioelectric signal recordings for diagnosis, assessment, or research work in collaboration with the specialized medical doctor. The neurophysiology technician has direct responsibilities of the application and of the result of the devices used. The required title is the 3-year degree (BS) in “Techniques of Neurophysiopathology.” The modern profile of the Neurophysiology Technicians should be not just an exam executor, but an expert specialist, completely integrated in the neurophysiological pathway [59]. Although sEMG analysis is already part of the neurophysiology technician background in general, sEMG procedures may be taught in greater depth with the purpose of generating professional figures capable of:

1. Perform all operations concerning sEMG data acquisition in accordance with established recommendations, consensus, and literature updates, including the proper use of devices.
2. Control the quality of sEMG recordings by detecting and correcting interferences and artifacts.
3. Perform sEMG signal processing and interpretation using commercial or home-developed software.
4. Understand the sEMG physiological and clinical correlates.
5. Perform initial and continuous patient assessments to ensure proper comprehension and compliance with testing methods.
6. Demonstrate the knowledge and abilities required to care for patients who require this monitoring mode.
7. Score sEMG activity and computer-generated results and maintain records to ensure comprehensive clinical information.
8. Comply with national and international documentation rules and submit a copy of the results to the physician.
9. Ascertain that adequate equipment and supplies are available to provide quality patient care, maintain inventories, and update reference materials.
10. Assist in the development and presentation of educational lectures, conferences, and promotional activities and act as point of contact for issues pertaining to sEMG studies.

The ability to properly detect and interpret sEMG and to avoid incorrect interpretations, for example in Gait Analysis [71], is certainly extremely relevant for different health professions. The European Curriculum in Neurorehabilitation, which is specifically addressed to medical figures (specialists in PMR, neurologists, etc.), highlights, in the didactic modules 2 and 3, that these figures must have knowledge of the tools for assessing muscle function including neurophysiological tests. Since in the Specialization courses such information is not included in the teaching activity, it is advisable to set up International or National Masters to fill these gaps [72]. A similar solution could be adopted also for physiotherapists, considering that usually they are not trained in the use of sEMG technologies, in a first-level Master degree open to both medical graduates and technical professionals. The certification by Scientific Societies is common in the US, but this method is not generally accepted in Europe [72], where, however, the possession of adjunctive degrees (first or second level master, PhD, etc) is a preferential or necessary qualification for some specific jobs.

### 4.3 New technical figures (clinical technologists)

Technical figures in the health delivery structures are not a novelty. Medical physicists have a fundamental role in using radiotherapy equipment, planning treatments, and processing images. Clinical engineers have been accepted as a general clinical figure for managing, maintaining, and using medical equipment together with physicians. “Laboratory Technologists” exist in Australian Departments of Physiotherapy since 1983 [73]. These figures are trained and hired to fulfill new needs resulting from technological developments not manageable by physicians because of the required knowledge, the lack, and high cost of their time.

Since 2003, the University of Twente and, since 2014, the Technical University of Delft, the University of Leiden, and the Erasmus University (The Netherlands) offer a BS in Clinical Technology and an MS in Technical Medicine (3 + 3 years). Up to 2022, over 600 students graduated, mostly finding jobs in public hospitals. See: https://www.tudelft.nl/en/education/programmes/bachelors/kt/bachelor-of-clinical-technology. The Dutch Association for Technical Medicine (NVvTG) was founded in 2009 as the professional association of a new healthcare professional, the Technical Physician (www.nvvtg.nl). See: https://www.tudelft.nl/onderwijs/opleidingen/masters/technical-medicine/msc-technical-medicine.

This activity has been presented as a task-shifting initiative, that is “passing the buck” of attaining/enhancing competences, work and responsibilities, from one professional group to another, as a remedy against medical workforce shortages, increasing impact of technology, as well as a way of lowering healthcare cost by shifting tasks from physicians to less costly personnel [74]. However, in the rehabilitation area, entirely new fields are developing such as neural engineering, sensors, signal processing, robotics, virtual reality, and many others. A specialized clinical technologist or technical physician could fulfill the demand for specific competence in the sEMG and related fields.

## 5 Who should teach what and to whom?

The need for updating the academic education of rehabilitation clinical operators has been extensively underlined in the literature and in the previous sections. Knowledge of the state of the art, including technology, is fundamental for posing and answering meaningful clinical questions. Many clinical questions were unthinkable before the decomposition of sEMG into its constituent MUAP trains was developed 20 years ago. Physiological knowledge, clinical questions, and technology are strictly inter-related [40,43,47]. Progress in the technical field has been so fast that even relatively recent textbooks become rapidly obsolete [75,76,77,78,79]. This fact requires frequent updates of academic courses that, unfortunately, have not yet been implemented. Therefore, what degree of technological background should clinicians have today to be able to communicate with their colleagues biomedical engineers and clinical technologists and apply recent developments? The issue has been discussed by McManus et al. [15,48]. A minimal set of fundamental concepts are listed below where the term “signal” applies to sEMG, to EEG, as well as to many biomechanical measurements. The following concepts should be translated into course contents and the basic ones should be taught at the BS level:

1. measurement, confounding factors, interferences, artifacts, noise [45,47];
2. mono-dimensional (1D), bi-dimensional (2D or images), and three-dimensional (3D or movies) signals evolving in time and space [7,16,80,81] (see Supplementary Material as examples of teaching complex concepts);
3. time and frequency representation of a signal: Fourier transform, amplitude and power spectra of a signal [8];
4. amplitude and frequency signal features [82];
5. signal diffusion in space and crosstalk between signal sources [83];
6. muscle fiber conduction velocity and its measurement [84,85];
7. decomposition of sEMG into its constituent MUAP trains [12,86,87];
8. neural drive to a muscle and its measurement [12,87];
9. input impedance, common mode rejection ratio, frequency response of a sEMG amplifier, signal sampling, and A/D conversion [8,10];
10. identification of reliable literature and familiarization with trustworthy resources in the field of sEMG; and
11. emphasis on checking equipment and procedures when using sEMG [88].

The above concepts are strongly interdisciplinary and is difficult to find teachers competent in all of them. In addition, this teaching requires extensive experience [26]. Although very simple textbooks are available in biomechanics [89] and sEMG [90] for non-engineers, their limited market is indicating their very limited use in schools. Praiseworthy efforts in teaching basic concepts to physiotherapists and movement scientists have been implemented in a 3 day free Winter School in Chile (29 physiotherapists, 18 from physical education, 2 from engineering, 75% of the time dedicated to signal processing) promoted by De La Fuente et al. [91]. See YouTube http://youtube.com/neuromechTV. In agreement with Jippes at al. [33], these authors “*believe that providing schools that are conducted 100% online could significantly benefit a larger number of students and professionals*” [92]. Related material, coming from academic lectures in English, is available at the URL https://www.robertomerletti.it/assets/pdfs/dr_sanjeev_gupta_video_lectures.pdf.

Other Summer Schools in closely related fields have been associated to the International Conferences in Neurorehabilitation [20,93,94] and free online tutorials are available from ISEK (https://isek.org/isek-jek-tutorials/).

Teaching regular academic in-presence lectures on these concepts requires 30–50 hours at the graduate level and 10–15 at the UG level, but this time could be reduced to half by using available online material. With a few exceptions, experienced clinical operators, trained many years ago (end even today) in the therapeutic application of heat, electromagnetic radiation, ultrasound, and electrical stimulation, would not be suitable teachers of the above concepts because of lack of competence. Biomedical engineers would likely lack the perception of the real clinical questions and needs. Neurophysiology technicians lack competence in the field of movement analysis and rehabilitation. The impact of clinical technologists has not yet been tested in this field. Therefore, a considerable effort is needed to combine “Teach the Teachers” initiatives, selection/preparation of good on-line material for home study, and discussion of recent literature in “journal clubs.” Availability of equipment for laboratory exercises, and teachers able to teach how to use this hardware and the related software, is a requirement that today is rarely satisfied. This is particularly the case in countries where Doctoral Degrees in physiotherapy are not available, a fact resulting in a vicious circle [10]. Considerable material is available on-line that can be used to support teaching (not to replace lectures). The issue of online *versus* in-presence lecturing [92] is quite interesting and should be seriously considered but exceeds the objectives of this work.

### 5.1 Target groups for sEMG knowledge translation and translation strategies

Many authors consider that sEMG teaching should take place at the MS level or at postgraduate Master courses. According to other authors, the main category to target for sEMG teaching is students of undergraduate (UG) programs. Many of these students will not pursue further studies and should therefore receive basic notions about sEMG at the UG level. The advantage with UG students is that they may reflect more keenness and curiosity toward sEMG practice. Students show interest in advanced instrumentation and their capacity to grasp technology is generally higher than that of practicing physiotherapists who are more oriented toward managing their patient loads. The natural tendency to resist change in clinical practices is less likely to be exhibited by the UG student community, especially if it is linked with curricular credits.

#### 5.1.1 Translation strategies

A three pronged strategy is necessary to introduce sEMG knowledge and instrumentation in the learner’s mind. The three prongs for such approach are (a) Educate, (b) Mandate, and (c) Reinforce. Educate: a thoughtful combination of appropriate theoretical concepts blended with practical sessions at para-clinical level of UG education is necessary to lay a strong foundation of sEMG practice and timely overlap of sEMG methods with related methods and protocols (e.g. biomechanics and IMU). This is the stage where the matrix of clinical practice is being laid in a budding clinician (learner) mindset. Mandate: the knowledge and application of sEMG should be made mandatory for clinical practice by statutory measures. It should include teaching and evaluation of sEMG knowledge with emphasis on quantitative reporting of muscle and movement features, sEMG clinical practice, student exchange programs (at the centers of excellence in sEMG), and attendance in seminars/conferences on sEMG. It should be made clear from the beginning of UG education that sEMG is an integral part of movement and treatment assessment. Reinforce: This is an unexploited area in sEMG education. Students should be posted as interns at sEMG laboratories as observers with limited but challenging tasks assigned to them. Fellowships, apprenticeships, academic document writing, supervised review of journal articles, and assisting teachers in preparation of notes and presentations are the non-conventional methods of teaching/learning that help the learners to reinforce their sEMG knowledge.

### 5.2 Required background for teachers and students enrolling in an sEMG course

The interdisciplinary nature of sEMG requires that sEMG courses should be offered at the second or third year of UG and at the graduate level to students who satisfy the minimal following requirements.

Minimal requirements for a student entering a sEMG course:

1. Clear concepts of basic physics and mathematics at senior secondary 12th grade standard level.
2. Understanding of basic neuromuscular anatomy and physiology required to apply sEMG theory and practice.
3. Capacity to overcome initially frustrating problems such as electrode placements, signal processing, feature extraction, parameter selection, and noise related issues.
4. Basic understanding to handle electrical instrumentation safely and diligently.
5. Basic understanding of computer software and digital platforms such as MS-Office, MATLAB, and similar.
6. Good knowledge of data banks, such as PubMed, and ability to search and read articles.

Minimal requirements for an sEMG educator/trainer:

1. Good knowledge and teaching acumen for principles of physics and mathematics related to biomechanics, electrophysiology, and biomedical signals.
2. Sufficient understanding of neuromuscular anatomy and physiology required to weave sEMG concepts into the students.
3. Experience with common clinical situations of movement disorders and use of sEMG to cite examples and relate to the learners quests.
4. Knowledge of common clinical practices of neuromuscular diagnosis and treatment such that an integrated teaching and assessment approach can be formulated.
5. Extraordinary communication and soft skills for the translation of mathematical concepts in a simple and comprehensible form.
6. Theoretical and practical knowledge of biomedical signal processing and MATLAB. Good supply of examples and exercises.
7. Full awareness of the sEMG capabilities and limitations. Familiarity with the literature.
8. Motivating and flexible personality to manage and steer learning goals in a problem-solving attitude suitable for the audience.
9. Ability to organize and manage a journal club.

These capacities are hard but not impossible to find among post-doctoral students and researchers working in existing research laboratories. The increase of the number of these figures of biomedical/rehabilitation engineers or life scientists with dual education must be promoted.

## 6 Conclusions and perspectives

There is a widespread consensus about (1) the need of reducing the gap between available knowledge in the field of rehabilitation assessment technology and the clinical application of such technology in physiotherapy and related fields, (2) the need to adopt EBP approaches to increase and quantify the quality of planning and evaluation of interventions and the effectiveness of therapies, and (3) the need to increase the competence and technical expertise of clinical operators in these fields. However, consensus on how to address these problems, how to form teachers, and how to disseminate knowledge and improve academic education, training, and competence of professional clinical operators is lacking. Consensus about a new educational curriculum for the next generation of clinicians is particularly urgent, not only because of the evolving technology but also because new technologies are changing our knowledge of physiology [47,95].

From the single channel recording and simple biofeedback applications of the 1980s, sEMG techniques evolved into the HDsEMG methodology, providing electrophysiological images and movies (similar to those of EEG), and are now addressing the clinical applications of decomposition of sEMG into the constituent motor unit action potential trains, the issue of EEG–EMG coherence, and the study of essential tremor, stroke, and other pathologies of the neuromuscular system. The current state of the art and the promising perspectives require a major updating of the competences of clinical operators in the interdisciplinary fields of biomechanics, biomedical signal processing, and neurophysiopathology. This competence is today available in research laboratories more often affiliated to Schools of Biomedical Engineering than to Schools of Medicine or Schools of Allied (or Technical) Medical Professions.

There is a strong need for simplification, standardization, and clinical versions of sEMG signal processing approaches and equipment that must be jointly developed by engineers and clinicians and then transferred to manufactures. A policy push to mandate sEMG as integral part of clinical practice in neuromuscular sciences is required to reinforce and update the learner’s knowledge and ensure its reflection in clinical procedures. A more concerted and transdisciplinary approach to aggressively reduce threats to sEMG acceptance should be initiated involving all stakeholders to reverse the current decaying situation. Few schools, training physiotherapists, and (in some countries) neurophysiology technicians teach sEMG technology and its clinical applications. The new figure of “clinical technologist” with strong interdisciplinary competences has been trained, at the BS and MS academic levels, and tested for 15 years in the Netherlands. Its impact in the rehabilitation field is still under evaluation. Discussion about the academic level at which courses on sEMG and other technologies should be offered (BS, MS, PhD, or postgraduate Master courses) is under way and is reflected in this work. Novel applications of sEMG in many fields (obstetrics, sport, ergonomics, gnathology, etc.) are rapidly developing and there is an urgent need to review the academic training of existing or new professional figures able to translate the available sEMG knowledge to these areas. There also is a strong need for internationalization of curricula among Higher Education Institutions, currently promoted by the EU and India project https://rishii-project.com/rishii-at-a-glance/.

The Supplementary Material provides two examples of support material for UG lectures dealing with the Fourier transform and the HDsEMG.

## Supplementary Material

Supplementary Figure
