# Supplementary material for: Translation of surface electromyography to clinical and motor rehabilitation applications: The need for new clinical figures
Source: Transl Neurosci. 2023 Mar 14;14(1):20220279. doi: 10.1515/tnsci-2022-0279 (PMC10024349; doi:10.1515/tnsci-2022-0279)
Supplement: Supplementary Figure [file tnsci-2022-0279-sm.pdf]

Supplementary material

The following Supplementary 1 and Supplementary 2 provide two examples of teaching two fundamental concepts (out of many) in sEMG without mathematical complexities. The first deals with the concept of Fourier transform, spectral analysis and filtering, the second deals with the concept of 2-dimensional (2D, images) and 3D (movies) sEMG. The interested reader can find more examples and free additional material under "Teaching material" in www.robertomerletti.it.

Supplementary 1: Teaching the fundamental concepts of the amplitude and power spectra of a signal with no mathematics.

This material provides an example of how a rather difficult mathematical concept, like the Fourier transform, can be explained in a relatively simple way, without mathematics, with limited loss of analytical rigor, to teachers who wish to teach the concept of sEMG spectrum.

Periodic signals

The concept of the Direct and Inverse Fourier Transform of a sampled signal is fundamental in sEMG analysis but mathematically difficult because it implies familiarity with the concepts of summation of the terms of a series, of complex numbers, of the imaginary unit ($i = \sqrt{-1}$), and of the complex exponential . Familiarity with these concepts cannot be expected by physiotherapy, movement science or neurophysiology students so a mathematical presentation cannot be used. The mathematical definition of direct and inverse Fourier transform is given in Equation S1 and S2 where $s(kt)$ is the sampled signal and $S(n/Nt)$ is its Fourier transform, t is the sampling interval and N is the number of samples of the signal segment (epoch) under consideration. It is therefore $t = 1/f_s$ where f_s is the sampling frequency of the signal, that is the number of samples taken per second.

Direct transform:

$$S\left(\frac{n}{Nt}\right) = \sum_{k=0}^{N-1} s(kt)\cdot e^{-i\frac{2\pi nk}{N}} \tag{S1}$$

where $n = 0, 1, 2, \ldots, N-1$ are the samples in time

Inverse transform:

$$s(kt) = \frac{1}{N}\sum_{n=0}^{N-1} S\left(\frac{n}{Nt}\right)\cdot e^{+i\frac{2\pi nk}{N}} \tag{S2}$$

where $k = 0, 1, 2, \ldots, N-1$ are the harmonics in frequency

While the above equations contain all the concepts related to the Fourier transform and to its properties, they are neither readable nor understandable by most life scientists and likely scare most physiotherapists and movement scientists. Despite the efforts of A. Samani in his praiseworthy book "Signal processing for non-engineers", CRC Press 2020, other approaches must be found to explain the concept in words and drawings, without mathematics, even at the price of a less rigorous presentation, as attempted in the example below. The example requires only the concepts of "signal", of "sinusoid", of "sampling", and of root mean square value (RMS) (described in slides 5 to 12 and 37 to 43 of teaching module 4 in https://www.robertomerletti.it/en/emg/material/teaching/module4).

Consider the red signal in Figure S1a. This is a periodic signal and two periods of 0.1 s duration are shown. This means that every 0.1 seconds the signal repeats itself and 0.1 s is its period. It can be demonstrated that *any* periodic signal, regardless of its shape and period, can be described as a summation of a number of sinusoids called "harmonics". Depending on the shape of the signal the number of harmonics required to build it up may range from a few to thousands. Note that each harmonic has its own amplitude and does not necessarily start from zero when $t = 0$. The red signal in Figure S1a is the sum of the four harmonics depicted in blue. These harmonics are mathematical building blocks and *do not* mean that the device or organ producing the signal actually produced them and added them up to create the signal. However, they fully describe the signal in time. Note that harmonic 1 has the same period (0.1 s) and frequency (10 Hz) of the signal, harmonic 2 has period half of that of the signal (0.05 s) and therefore double frequency (20 Hz), harmonic

3 has period 1/3 and frequency 3 times of the signal, and harmonic 4 has period ¼ and frequency 4 times that of the signal, and so on in case of more harmonics.

Let us now look at Figure S1a from the right and draw a green vertical bar indicating the amplitude of each harmonic at the corresponding frequency (A1 at 10 Hz, A2 at 20 Hz, and so on). We obtain a plot of amplitude of the harmonics versus frequency. This plot is called the amplitude spectrum of the signal. An example is given in Figure S1b for another signal having 10 harmonics. Each vertical bar represents the amplitude of one harmonic. For a reason given further below the amplitude spectrum does not depict the peak value of each sinusoidal harmonic (as in Figure S1) but its root mean square value (RMS), which is defined (only for sinusoids!) as $A_{\mathrm{RMS}} = A/\sqrt{2} = 0.707A$. The same procedure described above can be applied for the initial "phase" that is the angular value of the individual harmonics at time = 0.

A phase spectrum is obtained in this way but is not reported in Figure S1. The amplitude and phase spectra provide the representation of the signal "in the frequency domain" and contain the same information contained in the original signal (red curve in Figure S1a). They allow its perfect reconstruction. The reconstruction of the signal starting from its amplitude spectrum is the inverse Fourier transform of the amplitude spectrum. It is important to understand that these harmonics are mathematical entities and, in general, do not represent (and are not generated by) any physiological mechanism or phenomena. This process can be applied to any signal (voice, music, earthquake waves, sounds produced by whales or ship propellers, activity of sunspots, blood pressure wave, EEG, ECG, EMG, etc). Although the harmonics are NOT individually generated by the physical or physiological source of the signal they are extremely useful in the process of "understanding" the signal and "processing" it to extract physical or physiological information.

The power spectrum is obtained with the same procedure described for the amplitude spectrum but the height of the harmonics in the green part of Figure S1 represents the electrical power (instead of the amplitude) of the individual harmonics. For each harmonic of peak amplitude A this power is $A_{\mathrm{RMS}}^2 = A^2/2$.

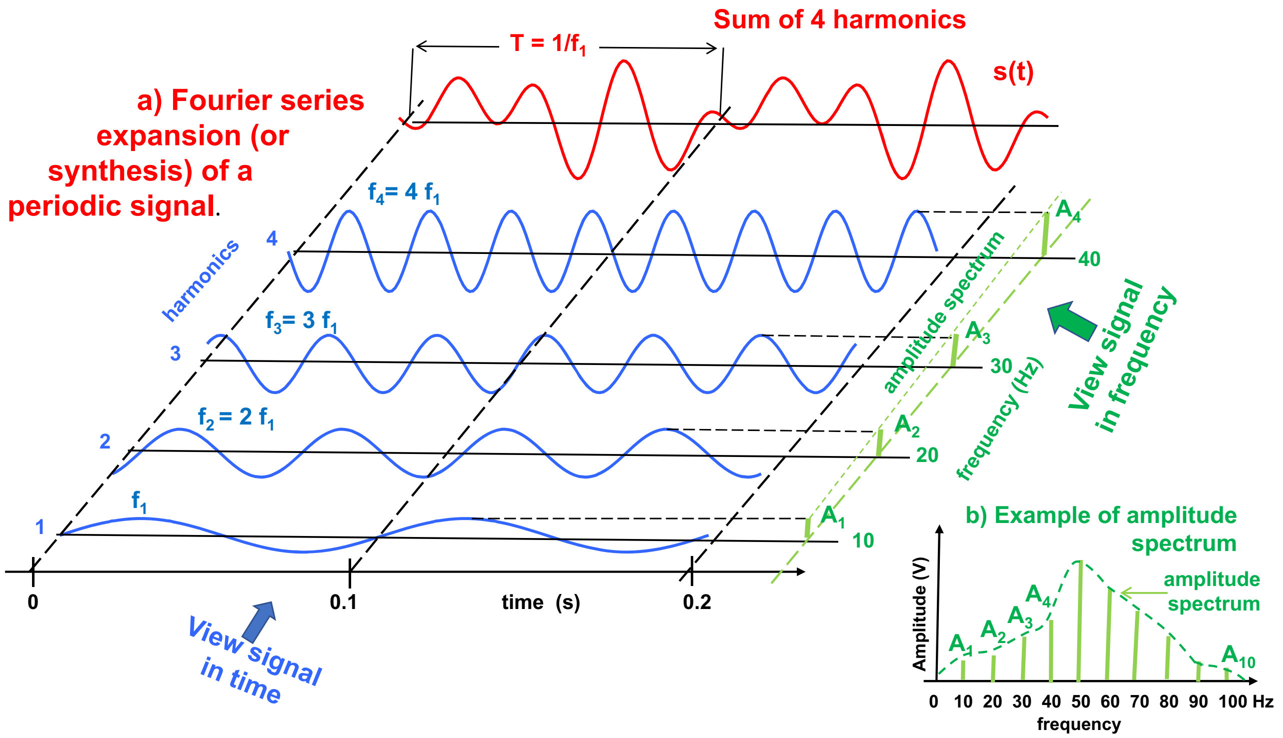

Figure S1: A sampled periodic signal with period of T seconds (0.1 s in the example) can be decomposed into the sum of sinusoids called harmonics (4 in this example) whose frequencies are multiple of $1/T$ (10 Hz in this example) as described in (a). The amplitude spectrum is obtained as indicated in green in (a) and in (b) for a signal having 10 harmonics. Note that these harmonics are mathematical entities and, in general, do not represent (and are not generated by) any physiological mechanism or phenomena. See text for further explanation.

Note that if the period T of the signal is longer, the harmonics will be closer to each other (because they are spaced by $1/T$ Hz) and if the signal is irregular and with fast changes the higher harmonics will have greater amplitudes than the lower ones.

Since the time and the frequency representations of a signal are perfectly equivalent, and one can be obtained from the other, what are then the reasons for a second representation? This question will be answered at the end of this section.

Stochastic signals (like sEMG or EEG)

Most bioelectric signals (including sEMG and EEG) are not periodic and appear to be random (stochastic), however, their analysis in the "frequency domain" (or frequency analysis" or "spectral analysis") can be performed with a mathematical "trick" consisting in taking a segment of the signal called "epoch" or "window" of duration T seconds and assuming that it repeats periodically with period T. Although this is not true, it allows a correct definition of the harmonics and of the signal amplitude and power spectra as outlined above for a periodic signal. Many closely spaced harmonics (separated by $1/T$ Hz) are usually required to obtain the spectra so that T cannot be too short.

Figure S2 shows an example concerning the frequency analysis of two different signals. The two signals are sampled (samples not indicated for clarity), have similar amplitude (and therefore similar RMS values), but are obviously different. One is "slower" and the other is "faster". How can we quantify this difference? The answer comes from the power spectra plots of the two signals (right panels), which are quite different: one is narrower and the other is wider. The harmonics, indicated by dotted vertical lines and separated by $1/T$ Hz in both cases, extend to different frequencies f_{max1} and f_{max2} and more power of signal 2 is associated to higher frequency harmonics than signal 1. This observation can be quantified in many ways. The most common is by means of the value of the centroid line (center of gravity or barycenter) of the power spectrum of the two signals. This is called *mean frequency*, it is a weighed mean, and should not be misunderstood as the mean of the frequencies from 0 to f_{max} (which is $f_{max}/2$). If we cut out the power spectrum of a signal on a piece of cardboard (red shapes in panel (b) and (d)) and we balance it on a blade indicated by the red dashed line, the piece of cardboard will be in equilibrium. This single number will give us some information about the width of the spectra. This is not the only indicator used and other features provide additional quantitative information about the difference between two signals and their spectra. More detailed explanation and examples of application are provided in slides 41 to 52 in https://www.robertomerletti.it/en/emg/material/teaching/module7.

Spectra and their mean frequencies (or other features) are similar but not identical when computed from subsequent epochs of a signal. For this reason they are called "estimates". This is a critical point when they are estimated

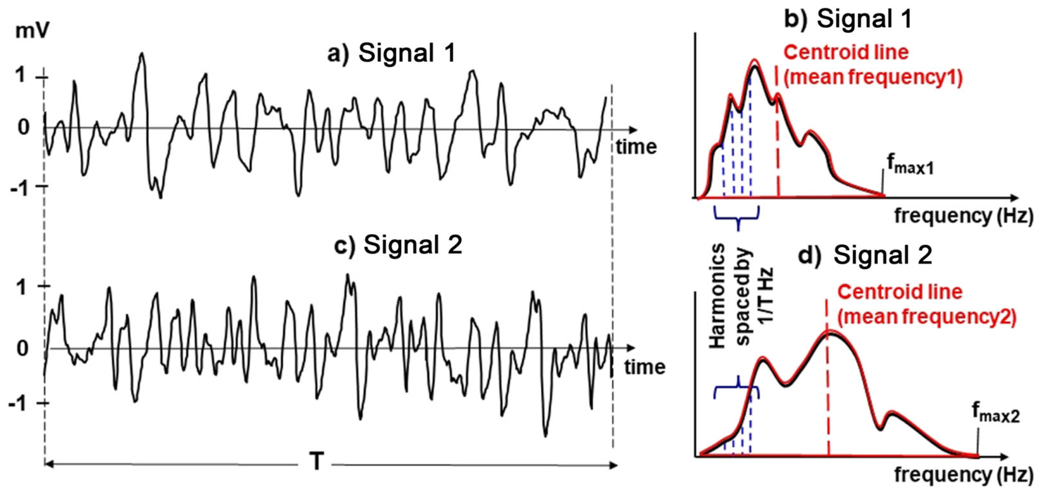

Figure S2: Example of two random signals (like sEMG) of similar amplitude but different power spectra. See text for further explanations. Signal 2 is the sEMG generated by a "fresh" muscle, signal 1 is generated by the same muscle at the end of a fatiguing constant force isometric contraction sustained to endurance time. The decrease of mean frequency is an indicator of fatigue.

from a signal that is changing its features in time, as the sEMG during dynamic contractions, and requires a careful choice of the epoch duration. Epoch duration (T) must be long enough to have a reasonable frequency resolution ($1/T$) and short enough so that the signal is reasonably "stationary" during each epoch. Proper choice is a compromise that depends on the velocity of shortening or lengthening of the muscle and the feature being computed. This requires some experience.

More on time and frequency domains. The concept of filtering

Although the time and frequency domain representation of a signal are equivalent, like two languages describing the same thing, the second allows operations on the signal that are very difficult to perform in the first. One such operation has been described above and provides a quantitative description of how "slow" or "fast" a signal is compared to another. Another important operations is filtering. Let us assume that we want to remove certain contributions to the signal provided by artifacts, power line interference, electrode-skin noise, etc.. This is not always possible but a way to do it, as a compromise, is the following:

1. Compute the amplitude spectrum of the signal (Figure S1b) by performing the direct Fourier transform (from the time to the frequency domain).
2. Remove (or reduce or modify) the amplitude of the undesired harmonics or group of them (if they can be identified).
3. Re-compute the time domain signal by performing the inverse Fourier transform (from the frequency to the time domain).

In this way a more "clean" signal is usually obtained.

More detailed explanations and examples of applications are provided in slides 30 to 34 in https://www.robertomerletti.it/en/emg/material/teaching/module4.

A relevant consideration concerns the choice of the epoch duration T. Since the $f_{\max}$ of the sEMG is is about 400 Hz, and the spacing between harmonics (frequency resolution) is $1/T$ Hz, the epoch duration must be chosen so that at least a few tens of harmonics are included in this frequency band in order to define the amplitude and power spectra with a reasonable resolution (spacing between harmonics). For example:

If $T = 0.1$ s; harmonics will be spaced by $1/T = 10$ Hz and the sEMG spectrum will be defined by about 40 harmonics. Estimates of spectral features may not be very good.

$T = 0.5$ s; harmonics will be spaced by $1/T = 2$ Hz and the spectrum will be defined by about 200 harmonics. There will be a more accurate representation of the spectral shape.

$T = 2.0$ s; harmonics will be spaced by $1/T = 0.5$ Hz and the spectrum will be defined by about 800 harmonics. A very good representation.

Longer epochs are critical if the sEMG signal is changing its features during one epoch duration. Shorter epochs are critical because the harmonics are further apart and the spectral definition is poor. The choice of epoch duration depends on the signal properties which depend on the test being done. Some experience is needed.

Applications

The analysis of sEMG in the frequency domain has many clinical applications not discussed here. A few are described in Ch. 4 and 10 of the book "Surface electromyography: physiology, engineering and applications" R. Merletti, D. Farina, IEEE Press and J. Wiley, 2016 and in the website: https://www.robertomerletti.it/en/emg/material/teaching/module10.

Particularly interesting recent research applications, based on spectral analysis, concern the study of tremor and of "coherence" between EEG and sEMG signals and provide information about the muscle control strategy by the CNS.

Supplementary 2. The surface electromyogram as a 2D or 3D signal

The purpose of this Supplementary is to show some of the many applications of two dimensional (2D, images) and 3D (movies) of the surface distribution of sEMG under an electrode grid.

A signal is the description of the values taken by a physical variable changing in time and/or in space and it implies the concept of measurement. Let us consider the following example. We measure the temperature on the wall of a room by means of a thermometer that is recording the temperature value in one spot during the day, that is in

time, either continuously (analog signal) or, for example, every minute (sampled signal at the frequency of 60 samples/hour). If the wall is partly in the sun and partly in the shadow its local temperature is not uniform and we can measure the temperature distribution in space, over the wall surface, with a number of properly placed thermometers, each providing a pixel of an image. For each instant of time we have an image of the wall region where the thermometers were placed and for subsequent time samples we have a movie where each set of simultaneous measurements in space is a frame. An image is a 2D signal (*x* and *y*) and a movie is a 3D signal (*x*, *y*, and *t*, that is a 2D signal evolving in time). We know that the temperature distribution in space is due to the infrared radiation from the sun being direct or shielded (by the blinds causing shadows) and we know that this distribution will change during the day because of the rotation of the Earth. This means that we “understand” the phenomena that are generating the signal and its changes in space and time, that is we have a “generation model”. We can “process” this signal and extract from it information about the temperature distribution, range or mean, the hour of the day, the cloudiness of the sky, the reflectivity of the wall, and/or make decisions about shape and position of the blinds causing the shadows and adjust them if needed. That means that we can build and apply a model for deeper understanding of the signal origin and properties.

The same applies to the sEMG distribution over the skin with much faster sampling. Thermometers are replaced by electrodes and temperatures by voltages. The nature and origin of the signal is very different with respect to the temperature example. Do we “understand” this signal? Do we have a model of it? What information can we obtain by “processing” it? Can this information improve our understanding and allow us to “adjust the blinds” in some ways? How, by whom, and for what purpose can this information be extracted and used? This Supplementary provides answers to some of these questions without any mathematics. A deeper understanding requires some knowledge of the mathematical algorithms used.

Consider the black part of Figure S3. Action potential trains from N α motor neurons drive N motor units (MU) that produce N motor unit action potential (MUAP) trains. These voltages appear on the skin and they add up algebraically in each point of the skin, in particular under Electrode #1, producing the voltage defined as EMG #1. The same happens under any other electrode placed on the skin. The red part of Figure S3 shows the same N MUs producing the same N trains of MUAPs that are now somewhat different because they are detected by

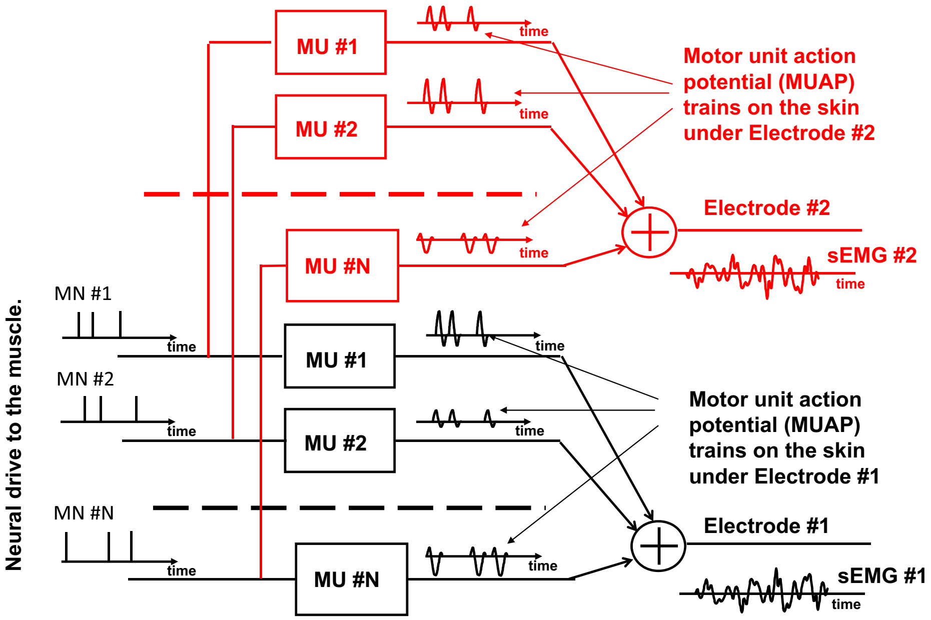

Figure S3: Current understanding of sEMG generation mechanisms from the neural drive to the muscle and from the muscle to the sEMG. The contributions provided in a particular point on the skin (e.g. under electrode 1 or 2) by the MUAP trains of N motor units are algebraically added to produce the monopolar sEMG in that point. This description is referred to as a “model”. See text for detailed explanation.

Electrode #2 which is in a different location on the skin and provides a different "point of view" with respect to Electrode #1. Therefore, the signal sEMG #2 will be different from sEMG #1. If we have M electrodes, placed in M different locations on the skin above the muscle, we will have sEMG #1, sEMG #2, sEMG #3..... sEMG #M providing M different points of view of ***the same*** N MUAP trains generated by *the same* N motor units. This technique is called "High Density sEMG (HDsEMG)" because the electrodes are "densely" placed. This is our current understanding of the sEMG generation mechanism deriving from decades of electrophysiology research. That is, we have a "model" of sEMG generation.

Can we use our understanding of the sEMG generation mechanism to process the sEMG signal(s) and extract the information contained in it and concerning the individual MUs? The answer is yes, with some limitations concerning : (1) the MUs should provide signals above the background noise level, so they cannot be too deep, and (2) a "sufficient" number of electrodes should be used to get the desired information. With proper software we can obtain information about the MUs (e.g. their conduction velocity, the location of their innervation zones, their length, their myoelectric manifestations of fatigue, etc.) and about the neural drive (the firing rate of each MU, the recruitment order, the "common drive" and the synchronization index of different MUs, the relations between the neural drive of agonist and antagonist muscle, etc. in physiological and pathological conditions such as fatigue, cramps, tremor, stroke, etc.). These sEMG processing techniques and the algorithms to obtain these results have been described in hundreds of scientific publications and a few textbooks. They are largely based on the "decomposition of the sEMG into its constituent MUAP trains", a method developed 20 years ago, whose application requires both a competence in physiopathology and in neuro-engineering as well as training and practical expertise in applying the best practices in the field and in the interpretation of the results.

A single pair of electrodes provides only a very small part of this wealth of information, like looking at a TV screen covered by a canvas with a single hole in it. In addition, the information provided depends on the position of the electrode pair over the muscle (like the location of the hole in the canvas). Teaching should first

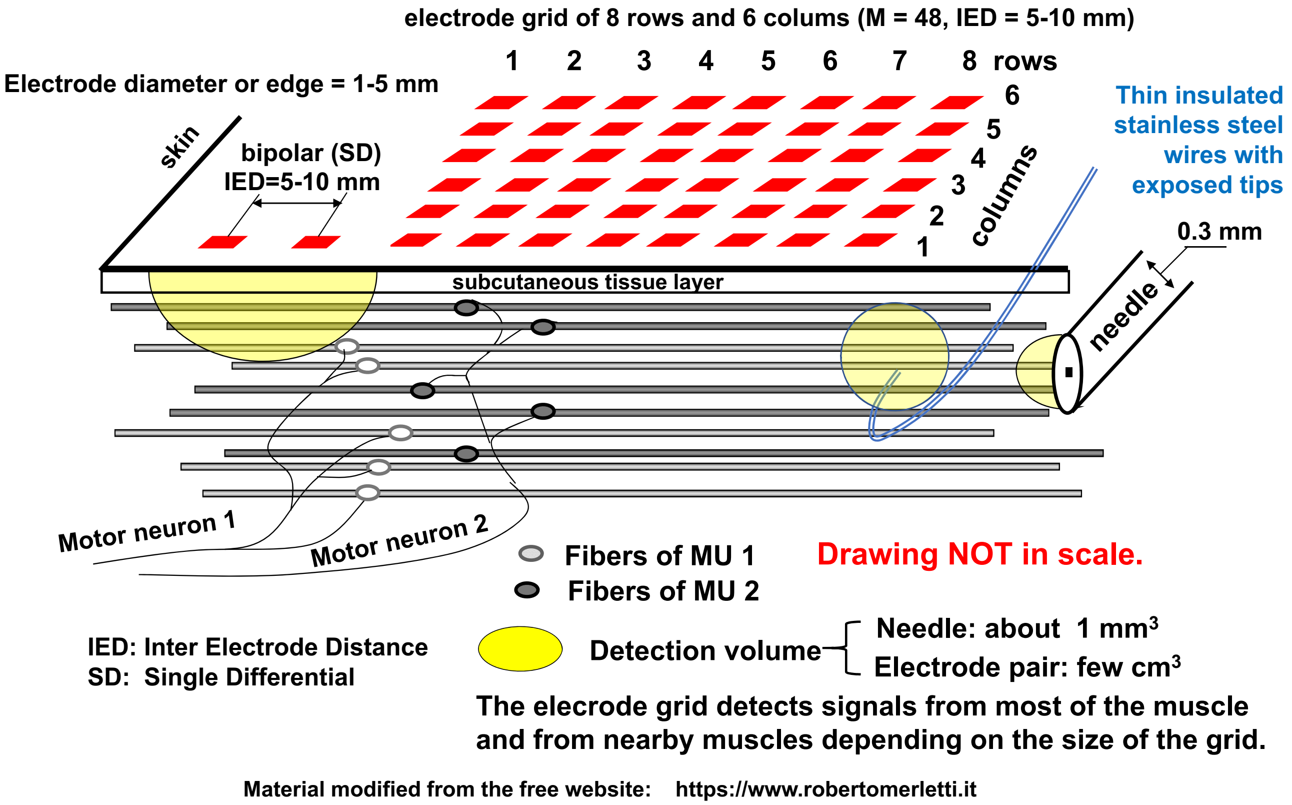

Figure S4: EMG detection systems. Four detection systems are described. From left to right: Bipolar detection, grid detection (HDsEMG), thin wire intramuscular detection, coaxial needle detection. The grid provides a spatially sampled image of the instantaneous surface distribution of the EMG signal. See also Figure S5 and videos in https://www.robertomerletti.it/en/emg/material/videos/.

introduce the concept of sEMG as an image (a "heat map") and then the special cases of monopolar or bipolar detection. Usually the opposite is done because the technological developments took place in the opposite direction, from monopolar or bipolar detection to HDsEMG.

Figure S4 describes the techniques available today for sEMG detection. We focus on the grid, or HDsEMG, which provides a time evolving image (a movie) of the sEMG amplitude distribution.

Figure S5 provides an example of images of instantaneous and root mean square (RMS) values over a given time epoch. For example, if the sampling frequency in time is 2000 samples/s there will be 2000 images/s or frames/s from which a movie can be obtained and displayed (as slow motion, off-line, to make it visible by the human eye). If the number of frames used for the RMS calculation and display is $N = 1000$ an RMS map (heat map) will be obtained every 0.5 s and a movie can be produced at the rate of two frames/s. The same concept applies to images of other quantities, such as the mean spectral frequency.

Figure S6 shows a sequence of RMS interpolated maps of longitudinal bipolar (single differential) signals obtained over a series of 20 1-s epochs from the two heads of the biceps brachii during a slow concentric and eccentric flexion and extension of the elbow while holding a 6 kg weight in the hand. A wealth of physiological information can be obtained from these maps, such as the role of the long and short head of the biceps during concentric and eccentric contractions, the recruitment and de-recruitment of motor units and their conduction velocity, the mean conduction velocity of superficial muscle fibers, the effect of slight supination/pronation of the forearm, etc. Note that the brachioradialis and brachialis muscles, playing a role in this task, are not monitored. An additional grid could be placed on the brachioradialis but detection from the brachialis is difficult because of its depth under the biceps.

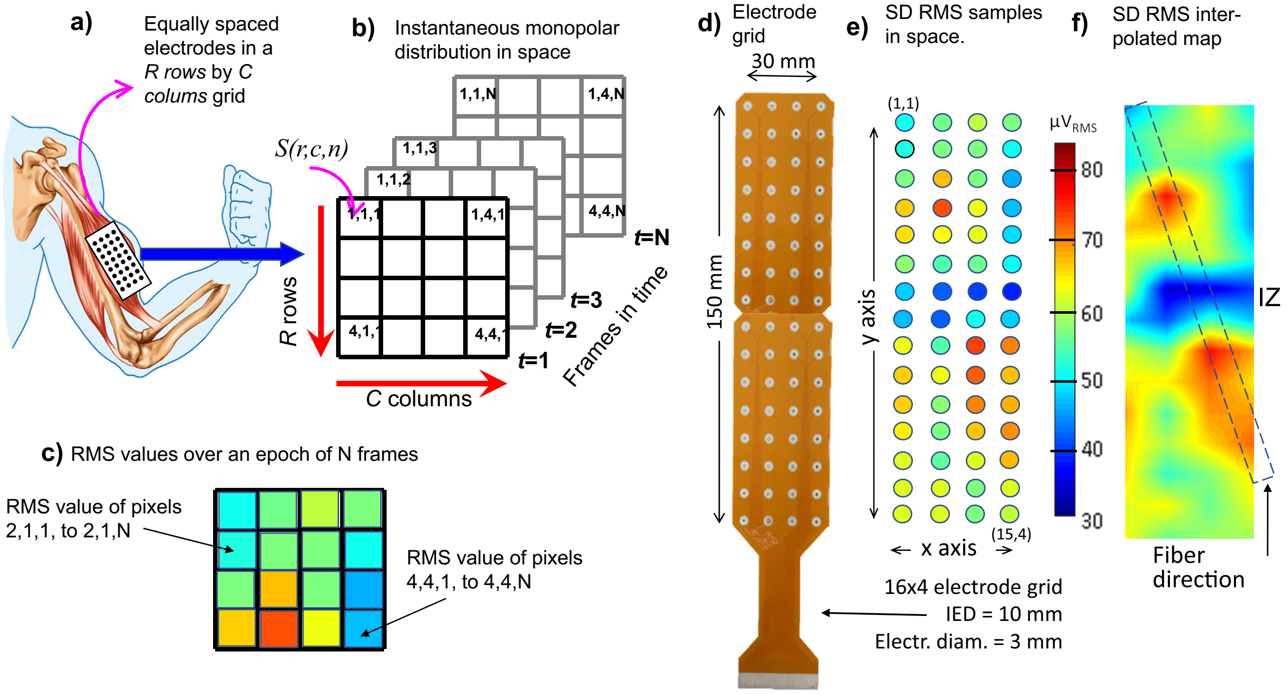

Figure S5: Example of HDsEMG detection. (a) grid placed on a muscle, (b) sequence of instantaneous frames: r = row number, c = column number, n = frame number, (c) RMS image computed over N frames from a 4 × 4 grid: one RMS frame is computed every N instantaneous images and a movie of RMS distribution can be obtained, (d) a 16 × 4 electrode grid, (e) one RMS map of bipolar (SD) signals obtained from the grid over 0.5 s, (f) one interpolated RMS map obtained from (e). The blue area is the innervation zone of the muscle. The color scale applies to both maps in (e) and (f). The dashed lines in (f) indicate the directions of the muscle fibers. Interpolation is meaningful only if the interelectrode distance (IED) is ≤10 mm. A few instantaneous and RMS movies can be seen in https://www.robertomerletti.it/en/emg/material/videos/.

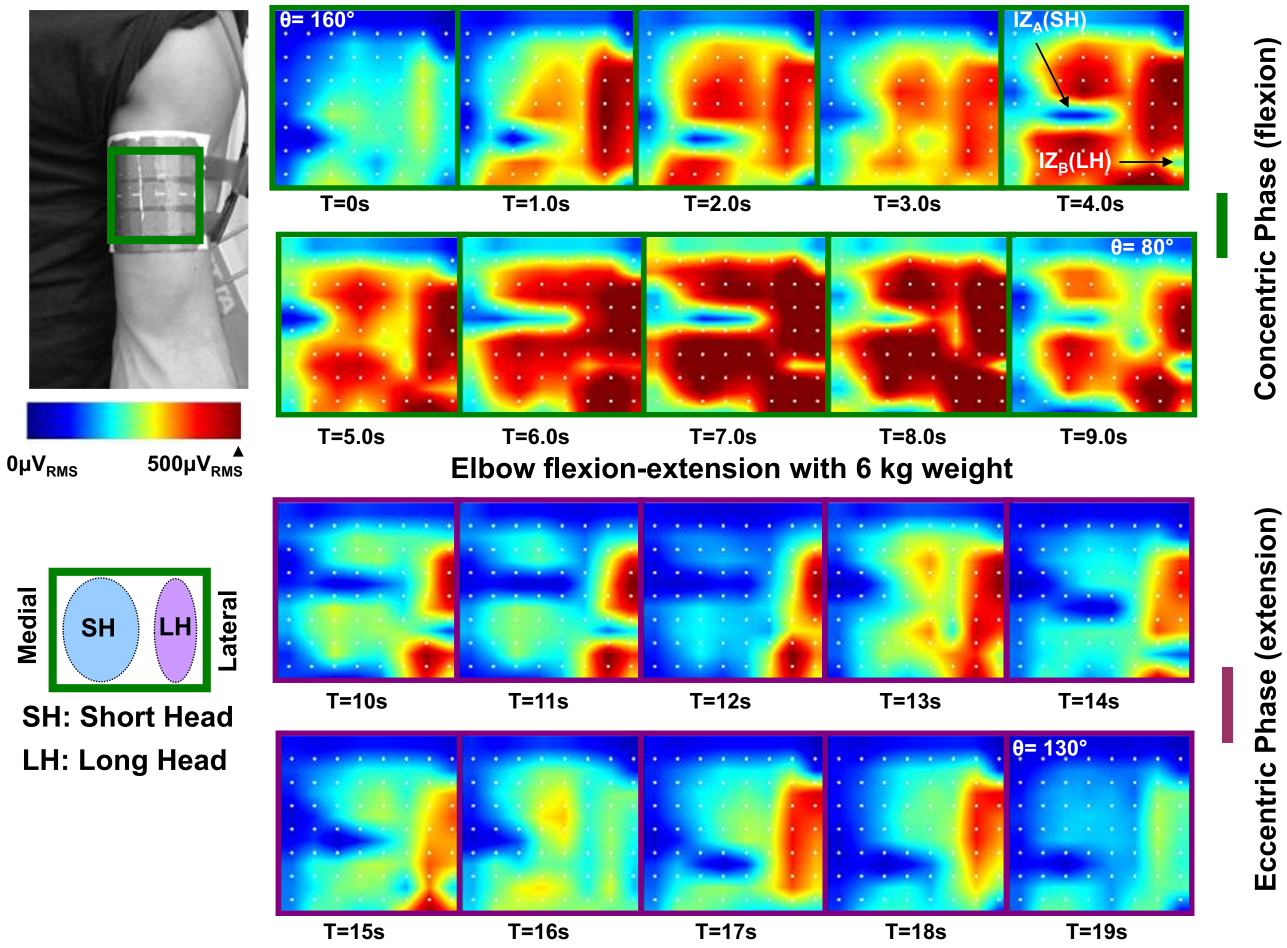

Figure S6: Sequence of RMS interpolated maps of longitudinal bipolar (single differential) signals obtained over 20 1-s epochs from the two heads of the biceps brachii during a concentric and eccentric flexion and extension of the elbow while holding a 6 kg weight in the supinated hand. Flexion and extension last 10 s each. $T=0$ means epoch from 0 to 1 s, $T=1$ means epoch from 1 to 2 s and so on. The white dots indicate the electrodes of the 8 × 8 grid used. A video of this task is available on https://www.robertomerletti.it/en/emg/material/videos/f10/.
